# Unraveling the Misconception About Deception and Nervous Behavior

**DOI:** 10.3389/fpsyg.2020.01377

**Published:** 2020-06-18

**Authors:** Aldert Vrij, Ronald P. Fisher

**Affiliations:** ^1^Department of Psychology, University of Portsmouth, Portsmouth, United Kingdom; ^2^Department of Psychology, Florida International University, Miami, FL, United States

**Keywords:** deception, non-verbal behavior, lie detection, cues of nervousness, illusion of transparency, perceived correlates of deception, actual correlates of deception

## Abstract

In this article, we attempt to unravel the misconception about deception and nervous behavior. First we will cite research demonstrating that observers believe lie tellers display more nervous behaviors than truth tellers; that observers pay attention to nervous behaviors when they attempt to detect deception; and that lie tellers actually feel more nervous than truth tellers. This is all in alignment with a lie detection approach based on spotting nervous behaviors. We then will argue that the next, vital, step is missing: Research has found that lie tellers generally do *not* display more than truth tellers the nervous behaviors laypersons and professionals appear to focus on. If observers pay attention to nervous behaviors but lie tellers do not come across as being nervous, lie detection performance is expected to be poor. Research has supported this claim. We finally discuss ideas for research into lie detection based on non-verbal behaviors.

## Unraveling the Misconception About Deception and Nervous Behavior

Distinguishing between truth tellers and lie tellers is an important task for a wide range of practitioners, including police officers, intelligence officers, and security personnel. It can be achieved through measuring (1) physiological responses, (2) brain activity, (3) non-verbal behaviors or (4) speech content. Of these methods, non-verbal lie detection is particularly popular, amongst other reasons, because it can be carried out all the time. It does not require equipment (needed for measuring physiological responses or brain activity) and does not require the target person to speak (needed for measuring speech content). The overwhelming view amongst practitioners (and laypersons) is that lie tellers display more nervous behaviors than truth tellers. In this article we provide evidence that this is a misconception, which could explain why people typically obtain poor accuracy rates when they make veracity assessments based on nervous behaviors.

We often refer to Mann et al. (2020) in this article, because the two experiments reported in that article demonstrate several of the points we want to make. See Appendix 1 for a synopsis of Mann et al. (2020) procedure and results relevant for the present article.

## Belief: Lie Tellers Display More Nervous Behaviors Than Truth Tellers

The belief that lie tellers display more nervous behaviors than truth tellers is well established. The most thorough investigation into beliefs about deception was carried out by Charles Bond (The Global Deception Team, 2006). Researchers from 58 countries collected data from 20 males and 20 females of their country. The participants were asked to answer the question: “How can you tell when people are lying?” They mentioned 103 different beliefs, four of which were given by more than 25% of the participants. Most people (64% of the participants) believed that lie tellers display gaze aversion and this belief was the most frequently reported in 51 out of 58 countries. The second strongest belief was “nervousness,” which was mentioned by 28% of the participants, followed by incoherent speech (25%) and body movements (25%). All four beliefs relate to nervousness and two beliefs (gaze aversion and body movements) relate exclusively to non-verbal behavior.

Apart from laypersons, also practitioners often associate nervous behaviors with deception (Strömwall et al., 2004; Vrij and Granhag, 2007; Vrij et al., 2018). In one study, 99 British police officers were asked to answer the question: “What verbal or non-verbal cues do you use to decide whether another person is lying or telling the truth?” (Mann et al., 2004). A total of 30 different beliefs emerged, of which two were mentioned by at least 25% of the police officers: Gaze aversion (mentioned by 73% of the police officers) and making body movements (mentioned by 25% of participants).

After the 9/11 terrorist attacks, the United States Transportation Security Administration (TSA) introduced SPOT (Screening of Passengers by Observation Techniques). In SPOT, trained individuals called Behavior Detection Officers (BDOs) observe passengers at airports with the aim to identify security threats. Cues that BDOs were taught to pay attention to included cues to nervousness such as avoiding eye contact, looking down, emitting a strong body odor, and covering the mouth with the hand when speaking (Denault et al., 2020).

The belief that lie tellers display more nervous behaviors than truth tellers also appears in police manuals (Vrij and Granhag, 2007). In these manuals deceptive behavior has been described as: Problem with eye contact, touching the nose, and restless foot and leg movements (Gordon and Fleisher, 2011); avoiding eye contact, frequent posture changes, grooming gestures, and placing hand over mouth/eyes (Inbau et al., 2013); rubbing the eyes, avoiding eye contact, and covering/rubbing the ears (Macdonald and Michaud, 1992); and moving the chair, abrupt and jerky behavior, problem with fine motor coordination, cold and clammy hands, using hands to cover mouth, and failure to maintain eye contact (Zulawski and Wicklander, 1993). See Vrij and Granhag (2007) for a more detailed discussion of the views expressed in police manuals.

Sometimes the beliefs of professionals and laypersons were investigated within the same study so that their answers could be compared directly (e.g., Akehurst et al., 1996; Vrij and Semin, 1996; Vrij et al., 2006). None of these studies found consistent differences among different groups of professionals; neither did the beliefs of professionals differ from those of laypersons. However, a different picture emerged for prisoners whose beliefs differed somewhat from the beliefs of both professionals and laypersons. Generally speaking, prisoners endorse the “lie tellers display nervous behaviors” beliefs less than non-prisoners do (Vrij and Semin, 1996; Granhag et al., 2004). For example, non-prisoners thought that deception is associated with more hand/finger movements, more trunk movements and more position shifts, whereas prisoners thought that such behaviors are not associated with deception (Vrij and Semin, 1996).

Finally, in two surveys, laypersons (Masip et al., 2012b) and law enforcement personnel (Masip et al., 2012a) completed a “beliefs about cues to deception” questionnaire based on the Behavior Analysis Interview, a lie detection method that relies, in part, on non-verbal cues of nervousness. It is popular amongst practitioners in many parts of the world (Inbau et al., 2013). The laypersons and law enforcement personnel expressed similar views (Masip et al., 2012a).

## Do People Pay Attention to Nervous Behaviors When They Try to Detect Deceit?

The belief that lie tellers display more nervous behaviors than truth tellers is relevant for lie detection, only if people actually pay attention to nervous behaviors when they try to detect deceit. In fact, they do. Vrij (2008) summarized the results of more than 30 studies analyzing the relationship between displaying nervous behaviors and being judged as deceptive. In those studies, truth tellers and lie tellers are videotaped and their non-verbal and verbal behavior is coded. Observers are shown those videotapes with the request to indicate after each fragment whether they think the person was telling the truth or lying. The observers’ judgments are then correlated with the target persons’ actual non-verbal and verbal cues displayed in the video fragments. The results showed that judged deception was associated with more gaze aversion and more movements (more fidgeting, hand/finger, leg/foot and trunk movements, shifting position) again suggesting that people pay attention to nervous behaviors when they try to detect deception.

A meta-analysis addressing the relationship between displaying nervous behaviors and being judged as deceptive showed similar results as Vrij’s (2008) review (Hartwig and Bond, 2011). Again, deception was associated with more gaze aversion, more fidgeting and more postural shifts. It was also associated with the general concept “nervousness,” a concept not examined by Vrij (2008)^1^.

In Mann et al. (2020) observers (laypersons) saw videotapes of laboratory experiments in which participants did or did not smuggle an object during a ferry-crossing. The observers were asked, amongst other questions, to indicate for each videotape whether the person was smuggling (yes/no) and to what extent the person came across as feeling nervous. Strong positive correlations between judging the person as a smuggler and perceiving the person as feeling nervous were found in Experiment 1 (*r* = 0.67) and Experiment 2 (*r* = 0.62)^2^.

## Do Lie Tellers Feel More Nervous Than Truth Tellers?

The implication of observers paying attention to nervous behaviors is that they think that lie tellers will feel more nervous than truth tellers. This assumption is supported. In Mann et al. (2020) the smugglers and non-smugglers were asked after completing their mission how nervous they felt. Smugglers felt considerably more nervous than non-smugglers in Experiment 1 (*d* = 0.73) and Experiment 2 (*d* = 0.62).

In a typical laboratory experiment, truth tellers and lie tellers are interviewed about an alleged experience. Sometimes they are asked afterward how nervous they felt during the interview. In his review of ten studies, Vrij (2008) concluded that lie tellers typically reported that they felt more nervous than truth tellers. Ekman (1985/2001; Ekman and Friesen, 1969) provided three explanatory mechanisms to account for lie tellers’ feelings of nervousness: Lie tellers, more than truth tellers, experience fear (of getting caught), guilt (of committing a morally disputable act) or duping delight (excitement of the opportunity to fool someone).

The finding that lie tellers generally feel more nervous than truth tellers does not mean that truth tellers do not feel nervous. They may feel nervous in interview settings, because they may experience fear (Bond and Fahey, 1987; Ofshe and Leo, 1997). Rather than experiencing fear of being detected (lie tellers), truth tellers may experience fear of not being believed because that could have serious consequences for them (further interrogations by police, taken aside by investigators at ferries or airports etc.).

## Do Lie Tellers Display More Nervous Behaviors Than Truth Tellers?

Both arguments presented so far – (1) observers pay attention to signs of nervousness when they try to detect deception and (2) lie tellers feel more nervous than truth tellers- work in favor of lie detection based on spotting nervous behaviors. However, one more step is required for such a method to work: Do lie tellers actually display more nervous behaviors than truth tellers? Only when lie tellers do so, can a lie detection method based on spotting nervous behaviors be successful. Research has found that lie tellers generally do *not* display more than truth tellers the nervous behaviors laypersons and professionals appear to focus on. A meta-analysis of deceptive behavior has shown that truth tellers and lie tellers display similar gaze behavior patterns and that lie tellers make *fewer* rather than *more* movements than truth tellers (DePaulo et al., 2003). This finding is sometimes challenged by practitioners or scientists who claim that these findings are based on laboratory-based studies where the stakes are low (Buckley, 2012; Frank and Svetieva, 2012). That is, there are for lie tellers no strong negative consequences associated with being detected or strong positive consequences associated with remaining undetected. They claim that in high-stakes situations the findings will be different. However, research does not support this claim. In their meta-analysis, Hartwig and Bond (2014) compared the behaviors displayed by truth tellers and lie tellers in (1) low-stakes situations and in (2) high-stakes situations. The same pattern of results emerged in both situations. It seems reasonable that lie tellers will display more nervous behaviors in high-stakes settings than in low-stakes settings; however, so will truth tellers, making the difference between the two groups unchanged.

In a truly high-stakes field-study, videotaped interviews with interviewees suspected of murder, rape, and arson were analyzed (Mann et al., 2002). Similar to the DePaulo et al. (2003) findings, the suspects showed no different gaze patterns when truth telling or lying, but they moved less when they lied. A selection of these videotapes was shown to police officers who were asked to indicate, amongst other factors, to what extent the suspects appeared nervous. Suspects who told the truth appeared more nervous than those who lied (Mann and Vrij, 2006). A similar finding was obtained in Mann et al. (2020). In Experiment 1, smugglers and non-smugglers made an equally nervous impression on observers, but in Experiment 2 smugglers made a *less* nervous impression than non-smugglers, representing a substantial effect size (*d* = 0.60).

Why are lie tellers more nervous than truth tellers, but are perceived by others as being equally or less nervous? In brief, we suggest that lie tellers actively attempt to alter their overt behavior to appear truthful (e.g., by minimizing signs of nervousness) whereas truth tellers are less concerned about others’ perceptions of them, and so they do not alter their behavior (Hocking and Leathers, 1980). In addition, lie tellers experience higher cognitive load in interviews than truth tellers (Vrij, 2014) and increased cognitive demand automatically reduces the amount of movements people make (Shallice and Burgess, 1994).

Both truth tellers and lie tellers believe their inner states shine through (Kassin, 2005; Granhag et al., 2007) and are knowable by others, the illusion of transparency (Gilovich et al., 1998). As a result, lie tellers cannot take their credibility for granted. They develop strategies to control their non-verbal behavior (Buller and Burgoon, 1996; Colwell et al., 2006; Hartwig et al., 2010) by attempting to avoid displaying behaviors they perceive as suspicious (Hocking and Leathers, 1980). In Mann et al.(2020, Experiment 2) two confederates approached the (non)smugglers on the ferry pretending to be looking for someone. This may have made both smugglers and non-smugglers nervous, but the smugglers –as a result for not taking their credibility for granted- may have tried to suppress displaying signs of nervousness more than the non-smugglers. Consequently, they made a less nervous impression on observers than non-smugglers. Attempting to control behavior is a mentally taxing strategy and could, as such, also automatically result in lie tellers making fewer movements than truth tellers and in a decrease in displaying nervous behaviors. Increased cognitive load leads to fewer hand and arm movements and inhibits fidgety movements (Ekman and Friesen, 1972; Shallice and Burgess, 1994; Ekman, 1997), because cognitive demand results in a neglect of non-verbal behavior, which subsequently reduces overall animation.

The previous and present section can thus be summarized as follows. Although lie tellers feel more nervous than truth tellers, lie tellers’ nervousness become less apparent in their behavior than truth tellers’ nervousness because (i) lie tellers actively try to avoid displaying signs of nervousness and (ii) cognitive demand automatically suppresses lie tellers’ expressions of nervousness.

## Accuracy in Lie Detection When Paying Attention to Nervous Behaviors

If observers pay attention to nervous behaviors but lie tellers do not seem to come across as nervous, lie detection performance is expected to be poor. That was indeed found in Mann et al.(2020, Experiment 2) in which the observers reported to have paid attention to nervous behaviors whilst the smugglers made a less nervous impression on observers than the non-smugglers (see above). The accuracy rate in distinguishing between truth tellers and lie tellers in that experiment was very low, 39.2%, which was significantly below the level of chance (50%).

Two more experiments addressed the relationship between paying attention to nervous behaviors and accuracy in truth/lie detection directly, both addressing the lie detection approach advocated by Inbau et al. (2013). In their manual, Inbau et al. (2013) reported that lie tellers display a variety of nervous behaviors, including gaze aversion, unnatural posture changes, self-self-adaptors, and placing the hand over the mouth or eyes when they speak. In Mann et al. (2004) police officers watched videotaped fragments of the real-life police-suspect interviews analyzed in Mann et al. (2002) and introduced above. Before starting the lie detection task, the police officers reported what they thought were indicators of deception. Results showed that the more “Inbau-cues” they mentioned, the worse they distinguished between truths and lies. In their experiment, Kassin and Fong (1999) informed half of the observers about the visual cues that Inbau et al. (2013) discussed in their manual. These trained observers performed worse on a subsequent lie detection test than untrained observers. Both studies suggest that paying attention to nervous behaviors identified by Inbau et al. (2013) as indicative of deceit hampers distinguishing between truth tellers and lie tellers. This is not surprising. Blair and Kooi (2004) examined the extent to which these “Inbau-cues” are identified as cues to deception in DePaulo et al. (2003) meta-analysis of the scientific literature. Little evidence was found in support of the Inbau-cues.

Most lie detection studies refer to non-verbal behavior in general rather than to nervous behaviors specifically. These studies show a bleak picture regarding non-verbal lie detection. A meta-analysis examining observers’ ability to detect truth and lies, showed an average accuracy rate of 52% in correctly classifying truth tellers and lie tellers when observers could only see (thus not hear) the target person, a percentage similar to chance level (Bond and DePaulo, 2006). Another meta-analysis examined the effect of training in non-verbal cues to deceit (Hauch et al., 2016). It revealed only a small positive effect.

## Reasons Why the Notion That Lie Tellers Will Display More Nervous Behaviors Exists

The notion that lie tellers will display more nervous behaviors than truth tellers appears to be a misconception. Yet, this notion remains popular. We think that at least three factors contribute to its popularity. First, a moral explanation (Bond and DePaulo, 2006). The belief that lie tellers avert their gaze and increase their movements fits well with the lying-is-bad stereotype. If lying is bad, lie tellers should feel ashamed (which leads to gaze aversion) and should be afraid of getting caught (resulting in gaze aversion and an increase in movements). Second, the accusation explanation (Vrij, 2008). Accusing someone of lying could easily result in a person displaying nervous behaviors out of fear not to be believed. Although this is likely to occur in both lie tellers and truth tellers, the interviewer may subsequently misattribute the suspect’s behavior to deception rather than to the accusation (Bond and Fahey, 1987). Third, the media exposure explanation (Hurley et al., 2014). There are many books [e.g., *Lie spotting* (Meyer, 2010) and *Spy the lie* (Houston et al., 2012)] and articles published in popular magazines or on the internet conveying the idea that lie tellers display non-verbal signs of nervousness. There is even a popular TV series “*Lie to Me*” about this idea. In other words, reading about deception or watching television could easily make someone think that nervous behaviors give lie tellers away.

## Is There a Future of Lie Detection Based on Non-Verbal Behaviors?

This article presented a pessimistic picture of lie detection based on nervous behaviors, making the future of this type of lie detection in our opinion bleak. However, this does not necessarily mean that lie detection based on non-verbal behavior in general has no future. There are arguments against and in favor of lie detection based on non-verbal behavior. The argument against is that four meta-analyses have shown detecting deception based on verbal cues to be superior to non-verbal lie detection. First, a meta-analysis examining observers’ ability to distinguish between truth tellers and lie tellers when observing target persons revealed an accuracy rate of 63% when observers could only hear the target person speaking, but an accuracy rate of 52% when observers could only see the target person and could not hear them speak, i.e., no verbal cues available (Bond and DePaulo, 2006). The accuracy when observers could both hear and see the target person was 56%. In addition, individual differences in the ability to distinguish between truth tellers and lie tellers seem to be minute (Bond and DePaulo, 2008). Second, a meta-analysis examining the verbal and non-verbal cues to deception revealed that verbal cues are more diagnostic indicators of deception than are non-verbal cues (DePaulo et al., 2003). Third, a meta-analysis examining the effect of training in verbal or non-verbal indicators of deception cues revealed a medium training effect for verbal lie detection training, but a small effect for non-verbal lie detection training (Hauch et al., 2016).

Two arguments can be made to continue non-verbal lie detection. First, perhaps future research will shed a more positive light on non-verbal cues to deception. Perhaps some non-verbal cues, yet unknown, will be found in the future that do reliably distinguish between truth tellers and lie tellers. Also, perhaps each lie teller gives his/her lies away in different ways (DePaulo et al., 2003; Levine, 2010; Levine et al., 2011). It will be challenging to identify the idiosyncratic pattern for each individual, but perhaps some general distinctions could show meaningful results. For example, there are individual differences in the frequency of lying (DePaulo et al., 1996; Hart et al., 2019) and perhaps frequent and infrequent lie tellers each display identifiable patterns of behavior that differ from each other. Perhaps signs of nervousness emerge in the infrequent lie tellers. Alternatively, some existing non-verbal cues may become diagnostic if they are examined differently. For example, Ekman (1985) has identified different types of smiles, including felt and false smiles. In an experiment, it was found that truth tellers displayed more felt smiles than lie tellers, whereas lie tellers displayed more false smiles than truth tellers (Ekman et al., 1988). Ekman’s best known deception work relates to micro-expressions of emotions that he claims lie tellers display: facial expressions that reveal a felt emotion and are suppressed within 1/5th to 1/25th of a second (Ekman, 1985). There is no evidence that micro-expressions of emotions distinguish truth tellers from lie tellers (Burgoon, 2018) or that training in observing such micro-expressions improves lie detection (Jordan et al., 2019). Finally, it is possible that, although no diagnostic cue to deception occurs when each non-verbal cue is examined individually, a diagnostic pattern will arise when they are examined in combination with each other (DePaulo and Morris, 2004). For example, in DePaulo et al. (2003) meta-analysis the impression of being tense was more strongly (albeit, in absolute terms, still weakly) related to deception (*d* = 0.27) than any of the individual non-verbal cues related to nervousness. Of these individual cues, frequency of pitch was the most diagnostic cue: *d* = 0.21 [see DePaulo et al. (2003), Table 6 and Table 8 cues based on a larger number of estimates]. It is unclear what the concept “impression of being tense” is made of; it is even not clear whether it contains non-verbal cues, verbal cues or a mixture of both non-verbal and verbal cues. Examining which individual cues contribute toward this concept is perhaps a venue for future research.

Second, sometimes relying on non-verbal cues may be the only lie detection option available, because target persons do not speak and their physiological and brain activity cannot be measured easily. An example is spotting potential wrongdoers in public spaces (airports, train stations, sporting events, concerts etc.). Lie detection in such situation can be very important, because national security can be at stake.

National security concerns are probably the reason why the SPOT program, introduced earlier in this article, has been introduced at United States airports. However, the U.S. Government Accountability Office (2017) recommended to limit SPOT funding due to lack of scientific empirical support for the cues BDOs rely upon. In this context, Denault et al. (2020) reported that SPOT is based on pseudoscientific claims, which could be attractive because (unlike scientific knowledge) they offer immediate and easy solutions to complex challenges.

Which behaviors practitioners should pay attention to at airports or similar settings is unknown because there is no scientific research available (Vrij et al., 2018). Research regarding non-verbal cues to deception almost exclusively concentrates on non-verbal behaviors displayed by interviewees in interview settings. People’s non-verbal behaviors are different when they are silent and walk rather than sit and talk. We therefore encourage researchers to examine non-verbal indicators of deception when other methods (such as speech) cannot be used. We do not expect this research to result in a clear-cut list of non-verbal cues that will identify wrongdoers. However, non-verbal lie detection may improve if the context in which the behaviors occur are taken into account, an approach advocated in verbal lie detection with promising results (Blair et al., 2010; Hartwig and Bond, 2011; Street, 2015). Another opportunity would be to increase behavioral differences between wrongdoers and others through -yet unknown- specific interventions (Hartwig and Bond, 2011).

## Author Contributions

RF raised the idea for the manuscript. AV drafted the manuscript. RF commented on the draft. Both authors contributed to the article and approved the submitted version.

## Conflict of Interest

The authors declare that the research was conducted in the absence of any commercial or financial relationships that could be construed as a potential conflict of interest.

## Footnotes

1 Hartwig and Bond(2011, Table 1) provide for 66 cues the actual and perceived relationship with deception, but they do not identify which of them are cues to nervousness.

2 The terms truth tellers and lie tellers refer to people who speak. Since the non-smugglers and smugglers did not speak we do not refer to them as truth tellers and lie tellers but they are the equivalent to non-smugglers and smugglers, respectively. That is, the notion that lie tellers experience more guilt, fear or duping delight than truth tellers (see the next section) equally applies to smugglers versus non-smugglers. Similarly, in the deception literature the terms *guilty* and *innocent* suspects are sometimes used. They are also the equivalent of lie tellers and truth tellers.

## Appendix 1

Outline of Mann et al. (2020). In two experiments, smugglers and non-smugglers made a ferry-crossing. Smugglers carried an object during the crossing; non-smugglers did not, they were told the experiment would commence on the other side after the ferry-crossing. The participants were secretly videotaped during the crossing. The difference between the two experiments was that in Experiment 2, two confederates approached the (non)smugglers (without talking to them) as they were searching for someone; no intervention took place in Experiment 1. After the ferry-crossing, smugglers in both experiments reported that they felt more nervous during the crossing than non-smugglers (measured on a 7-point Likert scales). The videotapes were shown to observers (laypersons) who were asked to indicate for each participant (i) whether they thought s/he was a smuggler or non-smuggler (dichotomous scale) and (ii) the extent to which they relied on signs of nervousness to decide this (measured on a 7-point Likert scale). First, positive correlations were obtained between judging someone as a smuggler and finding the person nervous. Second, in Experiment 1 the smugglers and non-smugglers were assessed as equally nervous but in Experiment 2 the smugglers were judged as being less nervous than the non-smugglers. Third, the correct classifications of smugglers and non-smugglers was at chance level in Experiment 1 (48.0%) but below chance level in Experiment 2 (39.2%).
